# Anxiety among Faculty Members of Academic Institutions of a Metropolitan City: A Descriptive Cross-sectional Study

**DOI:** 10.31729/jnma.8133

**Published:** 2023-04-30

**Authors:** Bimala Sharma, Nirmala Shrestha, Saurabh Kishor Sah

**Affiliations:** 1 Department of Community Medicine and Public Health, Gandaki Medical College Teaching Hospital and Research Centre, Pokhara, Kaski, Nepal

**Keywords:** *anxiety*, *faculties*, *prevalence*

## Abstract

**Introduction:**

Anxiety is one of the common mental disorders frequently occurring in the community. It has been a major contributor to public ill health. Very few studies have been conducted on anxiety among academic professionals working in educational institutions. The aim of this study was to find out the prevalence of anxiety among faculty members of academic institutions of a metropolitan city.

**Methods:**

A descriptive cross-sectional study was conducted among university faculties working in academic institutions of a metropolitan city from 22 July 2021 to 30 June 2022 after taking ethical approval from the Ethical Review Board (Reference number: 94). A self-administered structured questionnaire was applied to collect the information. Beck Anxiety Inventory was used to measure anxiety; the anxiety was categorised as normal, mild, moderate, and severe and dichotomized into "present" and "absent". Convenience sampling method was used. Point estimate and 95% Confidence Interval were calculated.

**Results:**

Out of 416 respondents, the prevalence of anxiety was found to be 111 (26.68%) (22.4430.92, 95% Confidence Interval). Among them, 85 (76.58%) were mild, 13 (11.71%) of moderate and 13 (11.71%) of severe type. Among those who had anxiety, 87 (78.37%) were males, and 59 (53.15%) were in the age group of 40 year and above; 37 (33.33%) had chronic health problems.

**Conclusions:**

The prevalence of anxiety among faculty members of academic institutions was lower as compared to other studies conducted in similar settings.

## INTRODUCTION

Anxiety is one of the common mental disorders occurring frequently in the community.^1^ Beck Anxiety Inventory (BAI) is a self-report measure of anxiety, widely used measure in both clinical and research settings.^2^

It is evident that anxiety is an important mental health condition in Nepal, and a major contributor to public ill health.^3,4^ The prevalence of anxiety was 16.1% among the adult population.^3^ Teaching is found one of the most stressful jobs.^5^ Job stress and working condition can lead to unpleasant emotions which impair academic professionals' ability to function or cope with daily life situations.

There was a lack of evidence on the prevalence of anxiety among faculty members working in higher academic institutions such as universities, campuses and colleges in Nepal. It is essential to generate baseline information on anxiety among them to initiate preventive measures.

The aim of this study was to find out the prevalence of anxiety among faculty members of academic institutions of a metropolitan city.

## METHODS

A descriptive cross-sectional study was conducted among faculty members teaching graduate and undergraduate students on the University, campuses and colleges in Pokhara Metropolitan of Kaski. The study was conducted from 22 July 2021 to 30 June 2022 after taking ethical approval from Ethical Review Board (Reference number: 94). Three public and three private/community academic institutions were selected. Convenience sampling method was used. The faculties who had been involved in the teaching profession as full-time faculties for at least one year and were assigned for teaching for at least bachelor-level education were included in the study. The faculties who were not involved in teaching jobs were excluded from the study. The sample size was calculated by using following formula:


$$\begin{array}{ll} \text{n} & ={\text{Z}}^{2}\times \frac{\text{p}\times \text{q}}{{\text{e}}^{\text{2}}}\\ & =1.96^{2}\times \frac{0.50\times 0.50}{0.05^{2}}\\ & =385 \end{array}$$

Where,

n = minimum required sample size
Z = 1.96 at 95% Confidence Interval (CI)
p = prevalence taken as 50% for maximum sample size calculation
q = 1-p
e = margin of error, 5%

The calculated sample size was 385. However, 416 samples were included in the study. A list of academic institutions was prepared; three public institutions (Prithvi Narayan Campus, Pokhara University and Pokhara Nursing Campus) and three private/community institutions (Janapriya Multiple Campus, Gandaki Medical College and Manipal Medical College) were selected. Numbers of full-time faculties working in the selected institutions were selected proportionately from each institution. If the selected faculty could not be contacted for consecutive two visits or was on long leave, the next faculty was selected as respondents from the same institution. Some faculties were found working in two institutions as different shift faculties; those repeated faculties were excluded from the list of the second institution.

BAI was used to assess the level of anxiety.^2^ BAI has a total of 21 items, each item has 4 options ranging from 0 to 3, 0 indicates not at all, 1 mildly, 2 moderately and 3 severely. Anxiety was categorized as BAI standard.^2^ Anxiety was classified into normal (0-7), mild (8-15), moderate (16-25) and severe (26-63); it was dichotomized into absent and present, present including all types of anxiety.^6^ Socio-demographic and behavioural characteristics were assessed using standard questions from previous studies.^7^ Behavioural characteristics included current smoking, current drinking, fruit consumption, vegetable consumption, physical activity and involvement in household work.

A standard questionnaire was prepared based on previous studies and standards.^2,7^ Adequate orientation was provided to the enumerators. The enumerators visited the institutions, met the selected faculties, provided the structured questionnaires and requested them to provide their information and experience in written form. Written informed consent was taken from the respondents. The filled questionnaire was collected on the same day or on the following days.

Data were entered in Microsoft Excel Version 2010 and analyzed using IBM SPSS Statistics version 21.0. Point estimate and 95% CI were calculated.

## RESULTS

Out of 416 respondents, the prevalence of anxiety was found to be 111 (26.68%) (22.44-30.92, 95% CI). Among those who had anxiety, 85 (76.58%) were mild, 13 (11.71%) of moderate and 13 (11.71%) were of severe type (Table 1).

**Table 1:** Prevalence of anxiety and its levels among faculties of academic institutions (n = 111).

Level of anxiety	n (%)
Mild	86 (76.58)
Moderate	13 (11.71)
Severe	13 (11.71)

Among the respondents who had anxiety, 87 (78.37%) were males, 73 (65.77%) were living in a nuclear family; and 106 (95.49%) were married (Table 2).

**Table 2:** Distribution of anxiety according to socio-demographic characteristics (n=111).

Variables	n (%)
**Sex**	
Male	87 (78.37)
Female	24 (21.63)
**Age (in years)**	
25-40	52 (46.85)
>40	59 (53.15)
**Family type**	
Nuclear	73 (65.77)
Others	38 (34.23)
**Marital status**	
Married	106 (95.49)
Unmarried	5 (4.51)
**Type of institution**	
Public	66 (59.45)
Private	45 (40.54)
**Education**	
Master	86 (77.48)
M.phil and PhD	25 (22.52)
**Work experience (in years)**	
1-5	20 (18.02)
6-10	26 (23.42)
>10	65 (58.56)
**Average monthly income (NRs)**	
<100,000	49 (44.14)
100,000-150,000	38 (34.24)
>150,000	24 (21.62)

Among those who had anxiety, 37 (33.33%) had chronic health problems (Table 3).

**Table 3:** Distribution of anxiety according to acute and chronic health problems (n= 111).

Parameter	n (%)
Having chronic health problem	37 (33.33)
Having health problem in last week	11 (9.90)

Of them who had anxiety, 30 (27.02%) consumed vegetables ≥3 times and 60 (54.06%) consumed vegetables only two times a day. Among those who had anxiety, 18 (16.21%) were smokers and 35 (31.54%) consumed alcohol ≥3 times in the last months (Table 4).

**Table 4:** Distribution of anxiety according to behaviour factors (n= 111).

Variables	n (%)
**Vegetable intake per week**	
<2 times a day	21 (18.92)
2 times a day	60 (54.06)
≥3 times a day	30 (27.02)
**Fruit intake**	
<1 time a day	19 (17.11)
1 time a day	67 (60.36)
≥2 times a day	25 (22.53)
Smoking in last 1 month	18 (16.21)
**Alcohol use in last month**	
Never	43 (38.74)
1 to 2 time	33 (29.72)
≥3 times	35 (31.54)
**60 minute PA per week**	
0 to 4 days	63 (56.75)
5 to 7 days	48 (43.25)
**Involvement in household work**	
Always/most of the time	42 (37.84)
Sometimes/rarely/never	69 (62.16)

## DISCUSSION

The study found that one in every four, i.e. 26. 68% had anxiety; of those who had anxiety, 11.71% had a severe type and the same proportion had a moderate type. In contrast to this study, a scoping review done on anxiety among teachers reported the prevalence of anxiety ranging from 38% to 41.2% which was higher than the prevalence of this study.^8^

In contrast to the study, the result of a systematic review conducted on the prevalence of anxiety in South Asia during COVID-19 showed that the pooled prevalence of anxiety in 31 studies was found to be 41.3%.^9^ Although the study population was different, the systematic review included 31 different studies conducted in South Asia. This shows that the prevalence of anxiety in the study was lower than the pooled prevalence of anxiety from previous studies. Higher than this study, the prevalence of anxiety was found to be 46.9% among undergraduate students of Pokhara Metropolitan.^10^ In contrast to the study, a study conducted among medical students and residents of a medical school in Nepal also revealed a higher prevalence of anxiety which was 45.3%.^11^ A review study done on the global prevalence and trend of anxiety among graduate students also showed that 34.8% of graduates suffered from anxiety.^12^ However, a study conducted among the adult population in Nepal shows a lower prevalence of anxiety which was 16.1%.^3^

Among those who had anxiety, the proportion of males was higher than the proportion of females in the study. However, as it was a proportion of males among total respondents with anxiety, it does not mean that problem was higher among males based on this research. Further analytical studies are required to test if sex influences the occurrence of anxiety or not. A study conducted in Nepal reveals that females had a higher likelihood of anxiety as compared to males.^3^ Some previous studies among different populations also showed age and sex influence anxiety status.^13,14^ Among the respondent with anxiety, 49 (44.14%) had monthly income less than NRs. 100,000 in the study. In line with findings, a Malaysian study revealed monthly household income was significantly associated with depression among patients with type 2 diabetes.^13^ A study conducted in Brazil also stated income influences the anxiety scores of participants.^14^

Some people who had anxiety also reported that they had chronic and current physical health problems. This shows that there might have been a concurrence of multiple health problems. This finding shows that having physical problems may influence the mental health status of people. In the study, among those who had anxiety, 35 (31.54%) respondents consumed alcohol three or more times in last month; and 18 (16.21%) smoked. A study from America found that regular smoker was almost twice as likely as occasional smokers to report a high level of depression as well as anxiety.^15^ This shows that harmful health behaviours not only influence physical health but also they can influence the mental illness.

Among those who had anxiety, 60 (54.06%) respondents ate vegetables two times each day in the study. A systematic review also recommends that the consumption of at least 5 portions of fruit and vegetables a day may be beneficial also for mental health.^16^ In the study, a higher proportion of respondents involved rarely or sometimes in the household work among those who had anxiety. In addition, 56.75% respondent with anxiety did at least 60 minutes physical activity for 0 to 4 days only in a week. Involvement in household work indicates physical activity and keeping oneself busy with some work.

The study has some limitations. As a descriptive cross-sectional study, it only provides the prevalence of outcome and proportion of categories of variables within the outcome. The study was conducted during the time of COVID-19 and clinical teaching faculties were also the study population. The situation might have overestimated the status of the outcome variable. As the study was conducted in Pokhara metropolitan, it may limit the generalization of the study findings.

## CONCLUSIONS

The prevalence of anxiety among faculty members was found lower than the other studies done in similar settings.
